# Supplementary material for: Bacterial Genes Outnumber Archaeal Genes in Eukaryotic Genomes
Source: Genome Biol Evol. 2020 Mar 6;12(4):282–92. doi: 10.1093/gbe/evaa047 (PMC7151554; doi:10.1093/gbe/evaa047)
Supplement: evaa047_Supplementary_Data [file evaa047_supplementary_data.pdf]

Overview of Supplementary information

Name	Content	SI	Website
SI Figure 1	Correlation of eukaryote genome size vs. number of homologous prokaryotic clusters	+	
SI Table 1a	Genome datasets (prokaryotes & eukaryotes), taxonomy, identifier, additional information		+
SI Table 1b	Prokaryotic sequence headers, sequence IDs		+
SI Table 1c	Eukaryotic sequence headers, sequence IDs		+
SI Table 2	Eukaryote-prokaryote clusters & eukaryote-specific clusters		+
SI Table 3	Numbers of assigned archaeal and bacterial homologs		+
SI Table 4	KEGG annotation count for archaeal & bacterial assignments		+
SI Table 5	Presence-absence matrix with organism sorting		+
SI Table 6	Archaeal, bacterial, eukaryote only clusters, clusters of whole prokaryotic clustering		+
SI Table 7	1,000 subsamples of bacterial datasets for assignment of prokaryotic origin of eukaryote sequences		+
SI Table 8a	KEGG sequences (v. September 2017)		+
SI Table 8b	KEGG BRITE hierarchy (v. September 2017)		+

Link to supplementary information on the website:

http://www.molevol.de/resources/index.html

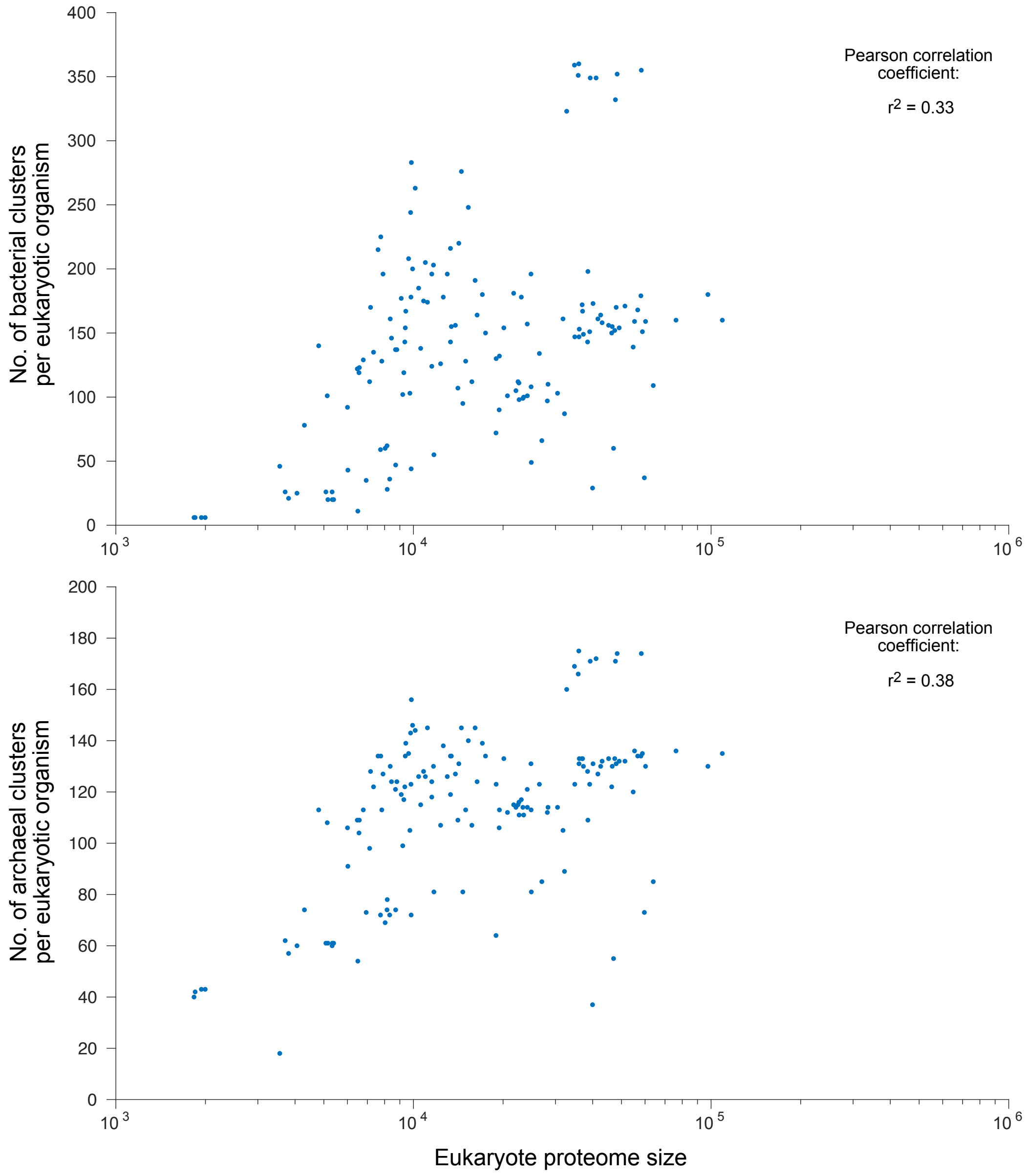

Supplementary figure 1: Correlation of eukaryote genome size vs. number of homologous prokaryotic clusters.
